# Genome-Wide Analysis of Basic Helix–Loop–Helix Superfamily Members Reveals Organization and Chilling-Responsive Patterns in Cabbage (*Brassica oleracea* var. *capitata* L.)

**DOI:** 10.3390/genes10110914

**Published:** 2019-11-08

**Authors:** Xi Shan, Wei Zhang, Fangwei Yu, Shenyun Wang, Jianbin Li, Jun Tang, Zhongliang Dai

**Affiliations:** 1 Zhenjiang Agricultural Research Institute, Jurong 212400, China; 2 Institute of Vegetable Crops, Jiangsu Academy of Agricultural Sciences, Nanjing 210014, China

**Keywords:** cabbage (*Brassica oleracea* var. *capitata* L.), bHLH transcription factor, chilling stress, RNA-Seq

## Abstract

Basic helix–loop–helix (bHLH) transcription factor (TF) family is commonly found in eukaryotes, which is one of the largest families of regulator proteins. It plays an important role in plant growth and development, as well as various biotic and abiotic stresses. However, a comprehensive analysis of the bHLH family has not been reported in *Brassica oleracea*. In this study, we systematically describe the *BobHLHs* in the phylogenetic relationships, expression patterns in different organs/tissues, and in response to chilling stress, and gene and protein characteristics. A total of 234 *BobHLH* genes were identified in the *B. oleracea* genome and were further clustered into twenty-three subfamilies based on the phylogenetic analyses. A large number of *BobHLH* genes were unevenly located on nine chromosomes of *B. oleracea*. Analysis of RNA-Seq expression profiles revealed that 21 *BobHLH* genes exhibited organ/tissue-specific expression. Additionally, the expression of six *BobHLHs* (*BobHLH003*, *-048*, *-059*, *-093*, *-109,* and *-148*) were significantly down-regulated in chilling-sensitive cabbage (CS-D9) and chilling-tolerant cabbage (CT-923). At 24 h chilling stress, *BobHLH054* was significantly down-regulated and up-regulated in chilling-treated CS-D9 and CT-923. Conserved motif characterization and exon/intron structural patterns showed that *BobHLH* genes had similar structures in the same subfamily. This study provides a comprehensive analysis of *BobHLH* genes and reveals several candidate genes involved in chilling tolerance of *B. oleracea*, which may be helpful to clarify the roles of bHLH family members and understand the regulatory mechanisms of *BobHLH* genes in response to the chilling stress of cabbage.

## 1. Introduction 

*Brassica oleracea* is widely grown all over the world, including cabbage (*B. oleracea* var. *capitata*), kale (*B. oleracea* var. *acephala*), Brussels sprout (*B. oleracea* var. *gemmifera*), cauliflower (*B. oleracea* var. *botrytis*), and broccoli (*B. oleracea* var. *italica*). *B. oleracea* is one of the most important leafy vegetable crops for studying morphological diversity and polyploidy evolution because of its unique U’s triangle theory and the *Brassica* lineage-specific whole-genome triplication (WGT) event [1]. The available genome sequences of *B. oleracea*, *B. rapa*, and *Arabidopsis thaliana* provided valuable information for plant improvement and biological research [1,2]. A large number of studies indicate that the regulations of plant growth and stress responses are coordinated by the combination of different network regulators, such as transcription factors (TFs). TFs can specifically recognize and bind the gene promoters in the nucleus, regulating the temporal and spatial expression of genes [3,4,5]. TFs respond to plant physiological, biochemical, and stress conditions by activating or inhibiting the associated downstream genes [5,6,7]. The basic helix–loop–helix (bHLH) TF is one of the largest families of TFs in plants [4]. The bHLH regions contain two functionally distinct regions, including the basic region and the HLH region [8,9]. The N-terminal is a basic region [10], which is mainly associated with the binding ability of the E-box (CANNTG) DNA *cis*-acting element. The C-terminal is a helix–loop–helix region, which includes two amphipathic α helices and one ring of variable length hydrophobic amino acids that are connected to form homodimers or heterodimers and to interact with other proteins. Nowadays, plenty of plant genomes are sequenced, a large number of bHLH proteins have been identified [4,6,7,11,12,13,14,15,16]. The bHLH proteins were divided into six major groups (A to F) through the phylogenetic relationship and specific protein motifs [17,18,19,20]. 

The bHLH proteins are involved in various signal transductions and metabolic pathways, such as light signal transduction [21,22], steroid signaling [23], jasmonic acid signaling [24], anthocyanin synthesis [25], and gibberellin synthesis [26]. Moreover, several bHLH proteins also regulate the formation of axillary meristem in rice [27] and the formation of root hairs [28]. Interestingly, several bHLH proteins can also respond to multiple stress conditions, like chilling [29,30,31], drought [32,33], salt stress [34], and iron homeostasis [35,36,37]. Under biotic and abiotic stresses, several bHLH proteins are activated and bind to the promoters of the target genes, then proteins are involved in signal transductions and stress responses. 

Chilling stress has a great effect on the metabolism and transcriptomes of plants. Chilling directly inhibits the enzymes of metabolism and reprograms the expression of genes [38]. ICE1 (inducer of CBF3 expression1)-CBF (CRT binding factor3)-COR (cold-regulated) chilling response pathway is one of the main signal transduction pathways for plant response and resistant to chilling stress. In *A. thaliana*, *AtICE1* encodes a bHLH TF, *AtICE1* can activate *AtCBF3* transcription by combining with MYC sites on the promoter of *AtCBF3* and regulate the response to chilling stress in *A. thaliana* [39,40,41]. ICE1, a CBF expression inducer, is an upstream regulator of chilling-responsive genes in various plants. ICE1-CBF pathway in diverse plants is conserved [42]. ICE1 can integrate different signals to adjust the chilling resistance of *A. thaliana* [43,44]. It has been shown that the protein kinase OST1 can phosphorylate and stabilize ICE1 under chilling stress, and increase the expression of the downstream chilling-tolerant genes [45]. However, mitogen-activated protein kinases (MPK3 and PMK6) reduce ICE1 stability and transcriptional activity by phosphorylate ICE1 protein, thus, negatively regulating the expression of CBF and chilling-tolerance of plants [40]. The transgenic analysis of different plants showed that the genetic engineering CBF pathway could significantly improve the chilling tolerance of plants. Overexpression of *ICE1* in cucumber [46] and rice [47] has been shown to enhance the chilling tolerance of the plants. It has been found that the *MdCIbHLH1* genes can be used to improve the chilling tolerance of the apple [48]. The bHLH family had been broadly studied in various plants. However, no systematic investigations on the bHLH TF family in *B. oleracea* have been reported. In this study, we comprehensively described the bHLH TFs in *B. oleracea* by comparative genomic analysis.

## 2. Materials and Methods

### 2.1. Sequence Retrieval in the B. oleracea Genome

The AtbHLH protein sequences of *A. thaliana* were collected from TAIR Database (http://www.arabidopsis.org/) and previous reports [9,11,49]. The BrbHLH protein sequences of *B. rapa* were collected from the *Brassica* Database (http://brassicadb.org/brad) [2]. To comprehensively identify the *BobHLH* genes, the annotated *B. oleracea* genome and protein sequences from the *B. oleracea* Genome Database (Bolbase, http://brassicadb.org/brad/index.php) were downloaded [1]. We searched the bHLH domain (PF00010) in *B. oleracea* proteome using the Hidden Markov Model (HMM) file with the E-values set ≤0.0001. The identified genes were submitted to the Pfam (http://pfam.xfam.org/) [49], SAMRT database (http://smart.embl-heidelberg.de/smart/batch.pl) and the online Batch CD-search tool (https://www.ncbi.nlm.nih.gov/Structure/bwrpsb/bwrpsb.cgi) [50], the redundant sequences were detected and the genes without bHLH domain were removed manually. The BobHLH protein sequences were named according to the position information in the chromosomes. 

### 2.2. Phylogenetic Analysis and Genomic Location of BobHLH Genes

Complete amino acid sequences of bHLH proteins were screened by the Pfam databases and identified the bHLH domains. MEGA 6.0 software [51] was used to construct neighbor-joining (NJ) distance trees with the bHLH proteins of *B. oleracea* (234 BobHLH proteins), *A. thaliana* (162 AtbHLH proteins) and *B. rapa* (230 BrbHLH proteins), the bootstrap values set 1000 replicates, which provided the reliability of the statistic. To localize the *BobHLH* genes on the chromosomes, the position information (starting and ending positions) of *BobHLH*s were collected from the *B. oleracea* genome, and the MapChart [52] software was used to show *BobHLH* genes on nine chromosomes.

### 2.3. The Expression Patterns of BobHLH Genes in Different Organs/tissues

To analyze the expression profile of *BobHLH* genes in different organs/tissues, RNA-Seq was downloaded from the NCBI GEO database (GSE42891), which contains the expression levels in seven organs/tissues (callus, root, stem, leaf, bud, flower, and silique). The expression abundance of *BobHLH* genes in different organs/tissues were calculated using the fragments per kilobase of transcript per million fragments mapped (FPKM) values. The hierarchical clustering heat map of the *BobHLH* genes was generated by using the pheatmap package (https://cran.r-project.org/web/packages/pheatmap/) based on the log_2_ (FPKM +1) values. 

### 2.4. Plant Materials, Growth Conditions, and Chilling Treat

The seeds of chilling-sensitive cabbage (CS-D9) and chilling-tolerant cabbage (CT-923) were germinated and grown in pots containing sterilized soil, the environmental conditions were set: 14 h light/10 h dark, 25 °C day/18 °C night. When the seedlings grew up to five true leaves, the seedlings were selected and transferred to the vernalization chambers for 6 h and 24 h under 4 °C chilling stress, while the non-treated seedlings (mock) were still under normal conditions. The young leaves from the chilling treated and non-treated seedlings were collected at 6 h and 24 h, respectively, three biological replicates were set at each time point, and immediately frozen in liquid nitrogen and used for RNA-Seq analysis. Finally, a total of 24 RNA-Seq libraries were constructed and performed on the Illumina HiSeq^TM^ 2500 platform. 

### 2.5. The Characteristics Analysis, Conserved Motifs, and Gene Structure of the BobHLH Genes 

Chilling-responsive *BobHLH* genes were selected to further study the characteristics analysis, conserved motifs, and gene structure. The theoretical isoelectric point (pI), amino acid number, molecular weight (MW), and hydrophobicity analysis of BobHLH proteins were analyzed by the ProtParam tool (https://web.expasy.org/protparam/). The conserved motifs of BobHLH protein sequences were identified by the Multiple Expectation-maximization for Motif Elicitation program (MEME, http://meme-suite.org/tools/meme) [53]. The parameters of the optimum motif width were set to 6 to 50 amino acids and the maximum motif number was set to 10. The Gene Structure Display Server (GSDS, http://gsds.cbi.pku.edu.cn/) [54,55] was used to draw the gene structure diagram according to the coding sequences and the corresponding genomic sequences of *BobHLH* genes. 

### 2.6. Characterization of Putative Cis-Acting Elements in the Promoter Regions of BobHLH Genes

The 2000 bp upstream of the translation start codon of chilling-responsive *BobHLH* genes were obtained from the *B. oleracea* genome. The *cis*-acting elements in the 2000 bp upstream regions of the chilling-responsive *BobHLH* genes were predicted by using the PlantCare database (http://bioinformatics.psb.ugent.be/webtools/plantcare/html/) [56].

### 2.7. Construction and Localization of Subcellular Localization Vector 

WoLF PSORT (http://wolfpsort.org/) [57] and BacelLo (http://gpcr.biocomp.unibo.it/ bacello/pred.htm) were used to predict the sub-cellular localization of BobHLH proteins. In order to investigate the sub-cellular localization of BobHLH proteins, we used the transient transformed GPF fusion expression vectors to transform the epidermal cells of tobacco (*Nicotiana benthamiana*) to observe the position of the fluorescent protein. Forward primers with *Sca* I restriction sites and reverse primers with *Xba* I restriction sites were designed, respectively (the specific primers were in the Table S10), cDNAs were used as templates for PCR amplifications, to obtain the full-length coding sequences of the *BobHLH054, BobHLH119,* and *BobHLH134* genes. The PCR amplifications were digested with *Sca* I and *Xba* I, then the fragments were ligated to the pCAMBIA2300-GFP vector. The constructed vector, as well as the empty vector (control), were transformed into *Agrobacterium tumefaciens* strain GV3101. *A. tumefaciens* were infiltrated into the tobacco leaves. After two days of infiltration, the GFP fluorescence was observed using a fluorescence microscope (OLYMPUS).

## 3. Results 

### 3.1. Identification and Phylogenetic Analysis of BobHLH Genes in B. oleracea

For the genome-wide identification of *BobHLH* genes, 234 available protein sequences were identified from the *B. oleracea* genome. It was confirmed that the 234 BobHLH proteins contain HLH domain. The 234 *BobHLH* genes were named from *BobHLH001* to *BobHLH234* based on their chromosomal positions (Table S4). 

**Figure 1:**
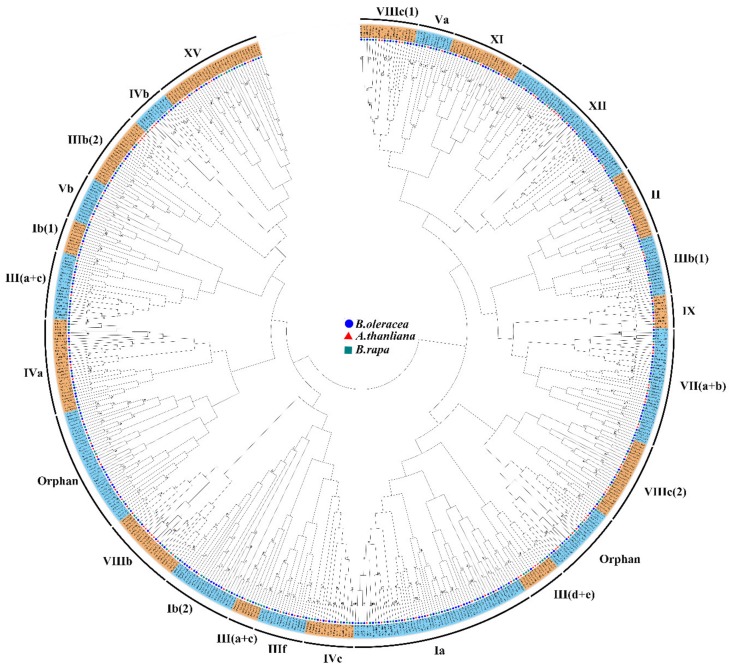
Phylogenetic tree of *B. oleracea, A. thaliana,* and *B. rapa* basic helix–loop–helix (bHLH) proteins. Phylogenetic analysis of bHLH proteins from *B. oleracea* (234), *A. thaliana* (159), and *B. rapa* (230) showing similar groups in the three species.

To study the classification and evolutionary relationships of BobHLH proteins, and to gain the potential function of BobHLH proteins for further investigation, the protein sequences of *B. oleracea*, *A. thaliana,* and *B. rapa* bHLH were used to generate a neighbor-joining (NJ) phylogenetic tree (Figure 1). *AT4G49770* (*AtbHLH95*), *AT4G38071* (*AtbHLH131*), and *AT2G20095* (*AtbHLH133*) were not found in *A. thaliana*. According to the clade support values and the classification of *A. thaliana* [11], the 234 BobHLH proteins were clustered into twenty-three subfamilies, while most of these subfamilies are common and have been reported in the phylogenetic trees of other species [6,12,13]. The results showed that the number of Ia subfamily member was the biggest and it contained 23 BobHLH proteins (Figure 1). III(d+e) subfamilies contained only two BobHLH proteins (BobHLH151 and BobHLH178). There were twenty-five BobHLH proteins that differed highly from the other subfamilies members and these were classified as “orphans.”

### 3.2. Chromosomal Localization of the BobHLH Genes in Cabbage

**Figure 2:**
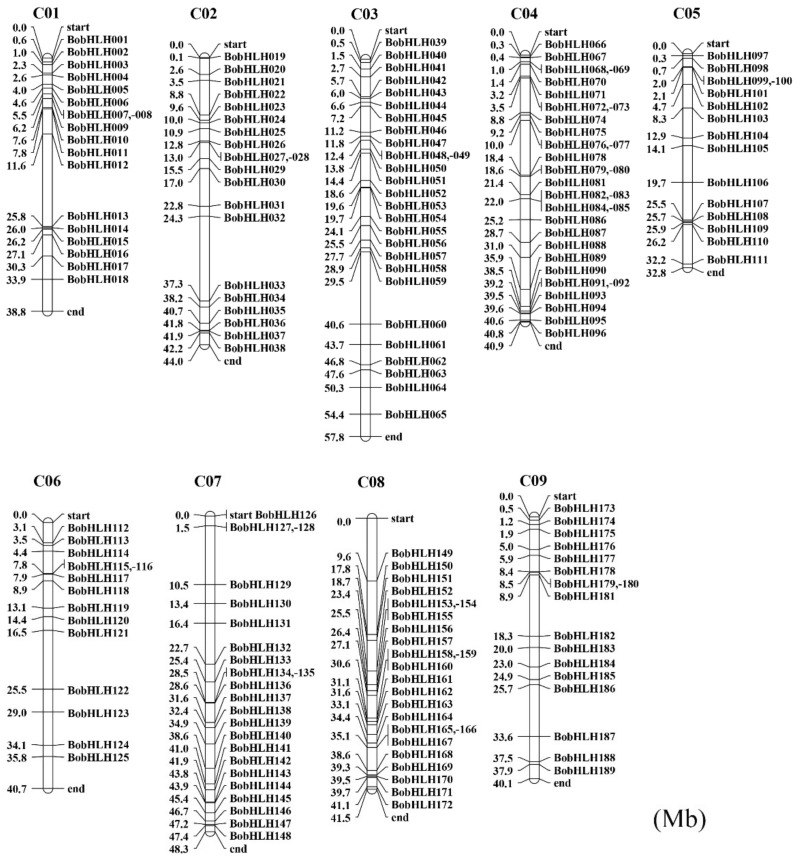
Distribution of *BobHLH* genes on nine *B. oleracea* chromosomes.

Based on the genomic position information, only 187 of the 234 *BobHLH* genes were unevenly distributed onto the nine chromosomes. The exact position of each *BobHLH* gene on chromosome (C01–C09) is shown in Figure 2. The rest of the 47 *BobHLH* genes were not anchored onto any of the *B. oleracea* chromosomes. Overall, the distribution of *BobHLH* genes was relatively dispersed, but some gene clusters also had relatively higher accumulation. Most of *BobHLH* genes were found on chromosome C03 (27, 14.4%) and chromosome C08 (24, 12.8%), while chromosome C06 (7.5%), chromosome C05 (8.0%), chromosome C09 (9.1%) had 14, 15, and 17 members, respectively. Several *BobHLH* genes were densely distributed on chromosomes regions, several chromosomes regions had no *BobHLH* genes distribution (Figure 2).

### 3.3. Expression Patterns of BobHLH Genes in Different Organs/Tissues 

**Figure 3:**
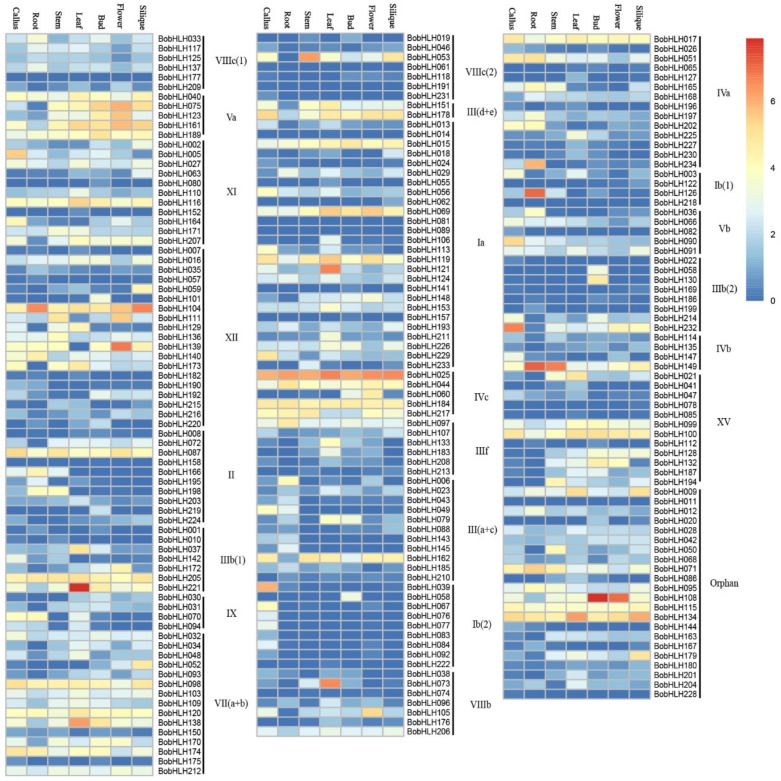
Expression profiles of *BobHLH* genes. Heat map representation of *BobHLH* genes in various organs/tissues, included callus, roots, stems, leaves, buds, flowers, and siliques. Expression levels of the *BobHLH* genes are shown as the log_2_ (FPKM+1), transformed FPKM values obtained from the RNA-Seq data.

To investigate the expression pattern of *BobHLH* genes, we used the FPKM values normalized data from RNA-Seq (GSE42891) and evaluated their expression patterns among seven organs/tissues. As showed in Figure 3 (and Table S5), the expression profiles of *BobHLHs* in callus, root, stem, leaf, bud, flower, and silique had significant differences. PA total of 197 *BobHLHs* expressed in more than two organs/tissues, the relative transcript abundance of three *BobHLHs* (*BobHLH081, BobHLH177, BobHLH218*) were 0, and 13 *BobHLHs* did not have transcriptional abundance data. Moreover, some *BobHLHs* showed unique tissue expression patterns, we found seven *BobHLHs* (*BobHLH022,* -*061,* -*076,* -*082,* -*083,* -*191,* -*228*) expressed only in the callus, *BobHLH154* was expressed only in the root, four *BobHLHs* (*BobHLH014,* -*055,* -*152,* -*222*) were specifically expressed in the leaves, six *BobHLHs* (*BobHLH 058,* -*089*, -*141,* -*157,* -*158,* -*219*) were expressed in the bud, and only *BobHLH011* was expressed in the flower. Two *BobHLHs* (*BobHLH062, BobHLH078*) were specifically expressed in silique. These results suggested that the specifically expressed *BobHLH* genes had special functions in specific organs/tissues.

### 3.4. Expression Analysis of BobHLH Genes in Response to Chilling Stress

**Figure 4:**
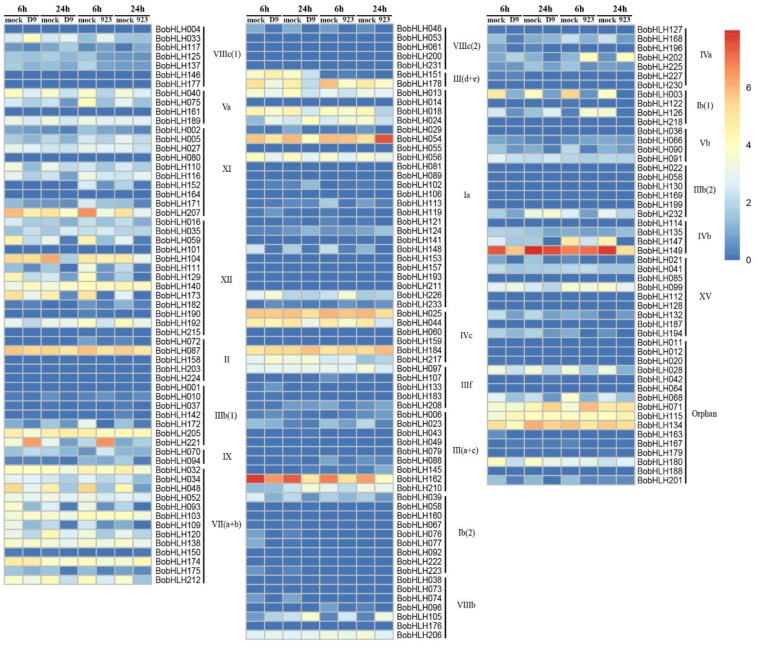
Expression profiles of *BobHLH* genes under chilling stress.

We also analyzed the expression patterns of *BobHLH* genes in CS-D9 and CT-923 under chilling stress (4 °C) and observed variation between the two different cultivars under different chilling stress (chilling for 6 h and 24 h; Figure 4; Table S6). A total of 181 *BobHLHs* were detectable in both of the two cultivars under chilling stress. There were several *BobHLHs* that have undetectable relative transcript abundance across the two cultivars under chilling stress (6 h and 24 h) and the mock-treated plants. When compared with mock-treated plants, three *BobHLHs* (*BobHLH033, -156,* and *-221*) were significantly up-regulated and seven *BobHLHs* (*BobHLH003, -048, -059, 093, -109, -129,* and *-148*) were significantly down-regulated in CS-D9 at 6 h chilling-treated plants. Compared with mock-treated plants, a total of eight *BobHLHs* (*BobHLH033, -070, -071, -105, -126, -156, -202,* and *-221*) and seventeen *BobHLHs* (*BobHLH003, -040, -046, -048, -059, -068, -075, -093, -109, -111, -129, -134, -148, -149, -172, -173,* and *-207*) were significantly up- and down-regulated in CT-923 at 6 h chilling-treated plants, respectively. At 24 h chilling-treated plants, there were three *BobHLHs* (*BobHLH045, BobHLH105,* and *BobHLH156*) and seventeen *BobHLHs* (*BobHLH003, -018, -039, -048, -054, -059, -093, -104, -109, -111, -147, -148, -162, -173, -178, -212*, and *-221*) in CS-D9, and six *BobHLHs* (*BobHLH054, -105, -117, -124, -156*, and *-202*) and nineteen *BobHLHs* (*BobHLH003, -018, -028, -044, -048, -059, -093, -094, -109, -111, -126, -129, -147, -148, -149, -152, -162, -173,* and *-212*) in CT-923 were significantly up- and down-regulated, respectively (Figure 4; Table S6). In CT-923, *BobHLH126* was significantly up-regulated in chilling-treated plants at 6 h under chilling stress, but the expression level was significantly down-regulated at 24 h. At the 24 h chilling stress, *BobHLH054* was significantly down-regulated and up-regulated in chilling-treated CS-D9 and CT-923.

### 3.5. Characteristics, Protein Motifs, and Gene Structures of BobHLH Genes

Sequence analysis showed (Table 1; Table S7) that the length of the BobHLH proteins varied widely. The molecular weight ranged from 10.25 (BobHLH112) to 109.54 kDa (BobHLH008). The protein length of BobHLH proteins ranged from 90 (BobHLH112) to 994 (BobHLH008) amino acids and the theoretical isoelectric point (pI) ranged from 4.49 (BobHLH185) to 10.26 (BobHLH188), a total of 91 BobHLH proteins was more than 7, and they were in the alkaline range, the BobHLH proteins were rich in basic amino acid. The average instability index of all BobHLH proteins was 56.93 (> 40.00), indicating that most BobHLH proteins were unstable. The analysis of the grand average of hydropathicity (GRAVY) showed that the value for BobHLH proteins was negative, which indicated that almost all the BobHLH proteins belonged to hydrophilic proteins.

**Figure 5:**
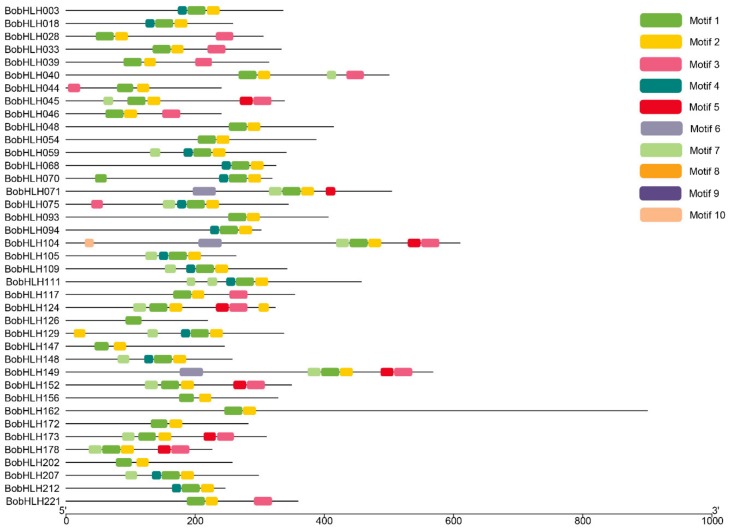
Chilling-responsive *BobHLH* protein motifs. The motifs are shown as colored boxes. The scale on the bottom may be used to estimate the length of the motif (unit: amino acid). The motifs in each BobHLH protein are in Figure S1.

To further analyze the diversification of BobHLH protein structure, the MEME program was used to predict the conserved motifs of BobHLH proteins (Figure 5, Figure S1). A total of ten conserved motifs were characterized by chilling-responsive BobHLH proteins (Figure 5), those of others were in Figure S1, and their logos were also generated (Figure S2). The species and numbers of these conserved motifs tended to be consistent with our phylogenetic tree results (Figure S1), several BobHLH proteins in the same subfamilies shared similar motifs, which means they might have similar biological functions [58]. Motif 1 and motif 2 were conserved in each BobHLH protein, except for BobHLH050 (only contains motif 1) and BobHLH143 (only contains motif 2; Figure S1). There were no motif 8 and motif 9 in the chilling-responsive BobHLHs (Figure 5).

**Figure 6:**
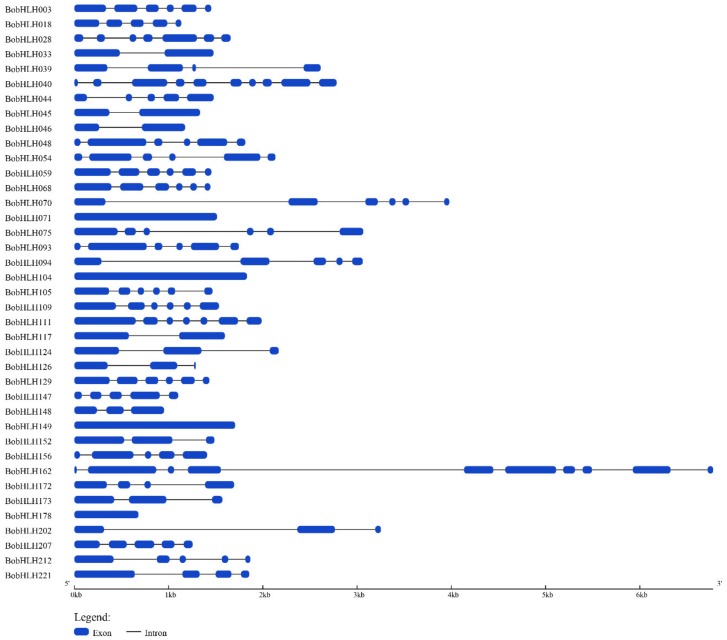
Gene structures of chilling-responsive *BobHLH* genes. The structures of *BobHLH* genes were plotted using blue boxes representing exons, black lines representing introns. The scale on the bottom is in the unit of kilobase (Kb). The gene structures in each *BobHLH* gene are in Figure S3.

Exon-intron structural diversity is considered to play an important role in the evolution of *bHLH* genes [59]. We analyzed the genomic DNA sequences and the corresponding coding sequences of 234 *BobHLH* genes to gain their exon-intron organization information (Figure S3). While only the gene structures of chilling-responsive *BobHLH* genes were shown in Figure 6. The others were shown in Figure S3. The same subfamilies shared a more similar gene structure including the number and length of exons and introns (Figure S3). The results indicated that the close evolutionary relationship in the same subfamilies [60]. Among the *BobHLH* genes, the exon numbers ranged from one to eleven. A total of twenty-five *BobHLH* genes had no intron, which accounted for 9.7% of the total *BobHLH* genes. The exon number of subfamily III(a+c) ranged from four to ten, while in subfamily Ia, it ranged from one to nine (Figure S3). The gain or loss of these exons may lead to the functional diversity of *BobHLH* genes.

### 3.6. Characterization of Stress-Related Cis-Acting Elements Among the Chilling-Responsive BobHLH Genes

**Figure 7:**
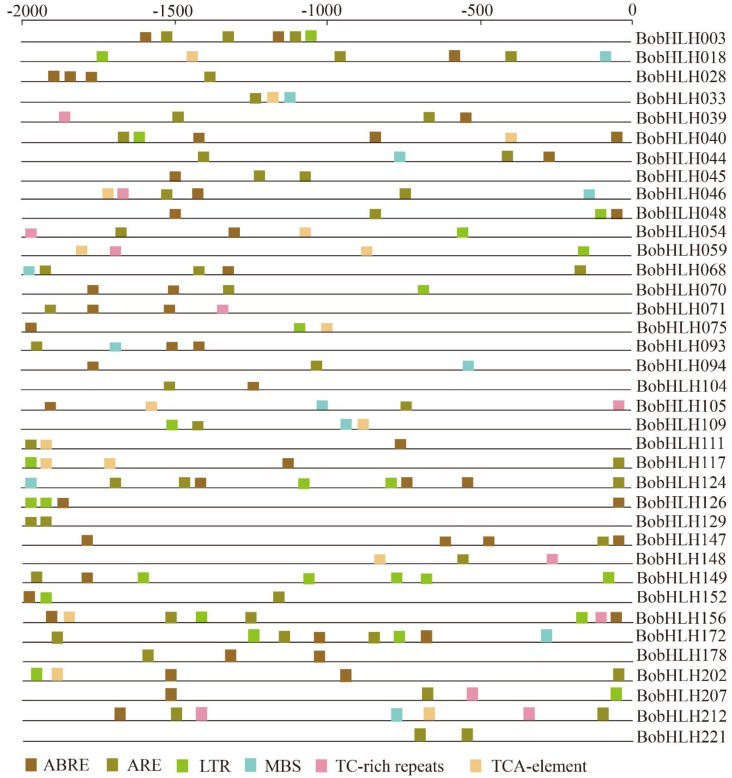
*Cis*-acting elements in the 2000 bp promoter regions of the chilling-responsive *BobHLH* genes.

The *cis*-acting elements of promoters are of great importance in response to abiotic stresses in the plant. The *cis*-acting elements from the transcriptional start site to the 2000 bp upstream regions were used to analyze their regulation mechanisms (Figure 7; Table S8), including ABRE (ABA-responsive element), ARE (anaerobic responsive element), LTR (low temperature responsive element), MBS (MYB binding site), TC rich-repeats and TCA-element. We investigated the *cis*-acting elements in chilling-responsive *BobHLH* genes (Figure 7), ABRE element plays an important role in ABA signaling and response to drought in *A. thaliana* [61], almost all of the *BobHLH* genes contained ABA-responsive element (except *BobHLH033, BobHLH059, BobHLH109, BobHLH148,* and *BobHLH221*). LTR element was found in eighteen *BobHLH* genes (*BobHLH003, -018, -040, -048, -054, -059, -070, -075, -109, -117, -124, -126, -149, -152, -156, -172, -202,* and *-207*), they had significantly different expression patterns in CS-D9 and CT-923 under chilling stress (Figure 4). This may be the reason they related to the chilling response [62]. It was reported that the MBS *cis*-acting element was binding to MYB transcriptional factors to involve stress signaling [63]; we found twelve *BobHLH* genes (*BobHLH018, -033, -044, -046, -068, -093, -094, -105, -109, -124, -172,* and *-212*) containing MBS.

### 3.7. Subcellular Localization Analysis of the BobHLH Proteins

**Figure 8:**
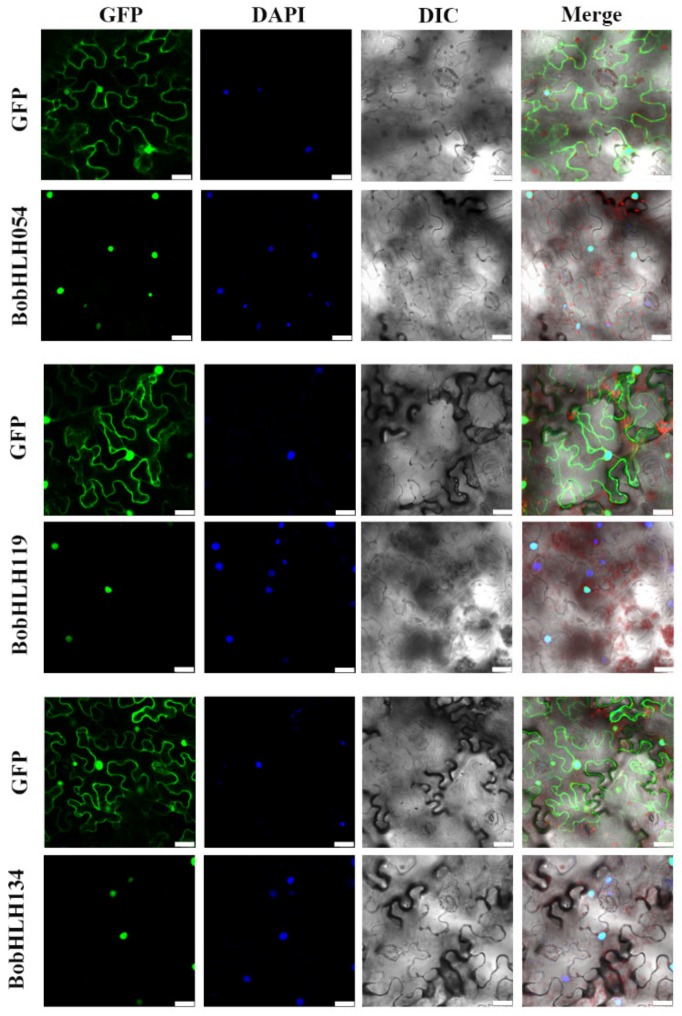
Subcellular localization analysis of BobHLH054, BobHLH119, and BobHLH134 in tobacco leaf epidermal cell. GFP stands for green fluorescence field, DAPI for DAPI field (nuclear staining), DIC for open field, Merge for superposition field. Excitation wavelength: GFP field: 488 nm, DAPI field: 358 nm, note that green fluorescence is the same as chloroplast spontaneous fluorescence excitation light and the wavelength of collected light is different. Bar = 25 μM.

WoLFPSORT was used to predict subcellular localization, the results showed that most of BobHLH proteins are present in the nucleus (except for the nineteen BobHLH proteins), while BaceLO predicted that 219 BobHLH proteins exist in the nucleus (Table S9), these predictions indicated that BobHLH054, BobHLH119, and BobHLH134 proteins are distributed in the nucleus. We observed that the fluorescence signal of the GFP signal was present on both cell membrane and nucleus in tobacco leaf epidermal cells (Figure 8), while the green fluorescent signal in epidermal cells transferred with BobHLH054-GFP, BobHLH119-GFP, and BobHLH134-GFP was concentrated in the nucleus, indicating that the three BobHLHs are nuclear proteins, this is consistent with the predicted results.

## 4. Discussion

The rapid development of plant genome sequencing has accelerated the studies of genomics and functional genomics, making it more convenient to identify and explore important genes on the whole genome level, while comparative genomic analyses have become more significant. The whole genome information is also used to explore the mechanisms of plant growth and development, the response to biotic and abiotic stresses. *B. oleracea* belongs to *Brassica* species and it is one of the most important leafy vegetables in the world. The completion of *B. oleracea* genome sequencing has given researchers more opportunities to characterize more gene families and to identify their phylogenetic relationships. bHLH TF is a large class of plant TF family, which play important roles in plant physiological metabolism and response to various stresses. 

In this study, we identified 234 *bHLH* genes in *B. oleracea* genome. In recent studies, the number of *bHLH* genes was varied; Chinese cabbage contained 230 *BrbHLHs* [12], 188 *MdbHLHs* had been identified in the apple “Golden Delicious” [64], and 113 *FvbHLHs* were detected in woodland strawberry [65]. The bHLH proteins classifications of several plants had been reported but the exact number is unknown. *A. thaliana* and rice are divided into twenty-six subfamilies and Chinese cabbage int twenty-four subfamilies [12]; according to the *A*. *thaliana* bHLH group nomenclature, bHLH proteins were separated into twenty-three subfamily in *B. oleracea* (Figure 1). These subfamilies were common in most species, suggesting that the bHLH proteins in conserved subfamilies might play an important role in plant evolution, while some non-conserved bHLH subfamilies may have evolved from plant-specific development or stress resistance [66]. Combined with the results of chromosome localization of *BobHLH* genes (Figure 4), several adjacent *BobHLHs* were clustered together on the same chromosome and they belonged to the same subfamily in the phylogenetic tree. *BobHLH013*, *BobHLH014*, and *BobHLH015* belonged to Ia (Figure 1) and they were clustered into chromosome C01 (Figure 4) while *BobHLH076* and *BobHLH077* belonged to Ib(2) and were clustered into chromosome C04. These results can also be observed in other plants [66], these clustered genes may share similar conserved molecular functions. The conserved motifs (Figure 5; Figure S1) and gene structures (Figure 6; Figure S3) of these clustered *BobHLHs* were similar. The results of predicted *cis*-acting elements of *BobHLHs* showed the Ia subfamily member *BobHLH013* contained LTR, while *BobHLH014* and *BobHLH015* did not. Usually, these clustered genes have some overlapping functions which might make them partially or totally redundant and lost [11].

According to the previous reports, *bHLH* genes were involved in stress responses. In general, the members of the same subfamilies may have similar molecular functions because of the specific conserved motifs, which could be used to predict the functions of unknown proteins. *B. oleracea*, *A. thaliana*, and *B. rapa* belong to *Brassica* and have close relationships, and the homologous genes among them were used to predict the functions in *B. oleracea*. It has been reported that *BrICE1* encodes a protein with the bHLH domain, which accumulates rapidly in response to chilling stress in non-heading Chinese cabbage [67]. *BrbHLH212* was expressed highly under chilling stress (12 h) in Chinese cabbage [12]. In this study, *BobHLH093* and *BrbHLH212* were clustered within subfamily VII(a+b) but it was significantly down-regulated expressed under chilling treat (6 h and 24 h), which may be due to its interaction with other bHLH proteins or non-bHLH proteins [11]. The homodimers or heterodimers formed between bHLH proteins or between bHLH and non-bHLH proteins determined the regulatory functions of bHLH proteins [62,68]. In the study of *A*. *thaliana*, *ICE1* and *ICE2* encode MYC-type bHLH TFs, and their orthologs were found to cluster into the same clade of subfamily VII(a+b) and III(d+e) with two corresponding members in *B. oleracea* (*BobHLH138* and *BobHLH151*), respectively. They increased the chilling tolerance by inducing CBF/DREB1 regulon and regulating stomatal formation [69], chilling-induction activated its post-translational modifications, including ubiquitination, phosphorylation, and sumoylation [43,70], so that the whole plant showed chilling resistance. The results of *cis*-acting element analysis showed that there was a number of defense-related *cis*-acting elements in the promoter region of *AtICE2*, which indicated that *AtICE2* might play an important role in plant biotic stress defense [71]. The promoter region of *BobHLH054* contained LTR *cis*-acting elements, which were involved in the chilling stress response, and it was significantly down-regulated in CS-D9, while it was significantly up-regulated in CT-923 at 24 h under chilling stress, indicating that an important role of *BobHLH054* gene in enhancing resistance to chilling stress. In the results of RNA-Seq, *BobHLH156* and *BobHLH003, -048, -059, -093, -109,* and *-148* were significantly up- and down-regulated, respectively. These *BobHLH* genes showed similar expression patterns. Genes with similar expression trends might interact at the protein level [72] and coordinate the regulation of downstream genes [4]. These mechanisms depend on the interaction of these genes and it is possible that the expression of one bHLH is affected by or regulated by another one, resulting in similar changes. Similar changes in their expression requires further study to explore the interrelationships between these genes. 

## 5. Conclusions

In this study, a total of 234 *BobHLH* genes were identified in the *B. oleracea* genome and were clustered into twenty-three subfamilies. Among them, 187 *BobHLH* genes were mapped to nine chromosomes, the rest were located on the scaffolds. Organ/tissue expression pattern analysis showed the organ/tissue-specific expression of *BobHLH* genes. RNA-Seq data analysis of chilling treat in *B. oleracea* indicated that compared with mock-treated plants, *BobHLH054* was significantly down-regulated in CS-D9 and up-regulated in CT-923 at 24 h under chilling stress, which means that it may be involved in the chilling-response. The molecular weight of the BobHLH proteins ranged from 10.25 to 109.54 kDa. The characterization of the proteins and genes, including conserved motifs and gene structures, were similar in the same subfamilies. The stress-responsive *cis*-acting elements analysis showed that several of chilling-responsive *BobHLHs* contained LTR in the upstream regions. These findings in this study provide a foundation to investigate the information of *BobHLH* genes including the candidate genes of *B. oleracea* against chilling stress for further functional studies.

## Figures and Tables

**Table 1:** Chilling-responsive *BobHLH* genes in *B. oleracea*.

Gene Name	Gene ID	Subgroup	MW (kD)	PL (aa)	pI	Instability Index	Aliphatic Index	GRAVY
*BobHLH003*	*Bol013674*	Ib(1)	38.02	336	5.23	50.35	62.89	-0.58
*BobHLH018*	*Bol034680*	Ia	29.33	258	7.09	43.18	69.53	-0.61
*BobHLH028*	*Bol028622*	Orphan	33.56	305	5.66	59.74	74.20	-0.79
*BobHLH033*	*Bol031047*	VIIIc(1)	38.22	333	6.11	56.65	70.30	-0.80
*BobHLH039*	*Bol015349*	Ib(2)	35.87	314	6.20	54.79	81.11	-0.56
*BobHLH040*	*Bol008832*	Va	55.21	500	7.25	64.06	59.86	-0.86
*BobHLH044*	*Bol027942*	IVc	26.23	240	9.08	57.23	62.62	-0.69
*BobHLH045*	*Bol028008*	Ib(1)	37.16	338	5.59	77.92	85.65	-0.53
*BobHLH046*	*Bol020475*	VIIIc(2)	26.70	240	7.60	51.58	80.92	-0.41
*BobHLH048*	*Bol029437*	VII(a+b)	46.22	414	5.75	64.00	57.22	-0.83
*BobHLH054*	*Bol010742*	Ia	42.56	387	6.80	66.78	59.30	-0.64
*BobHLH059*	*Bol012482*	XII	38.43	341	5.89	62.00	74.57	-0.61
*BobHLH068*	*Bol004843*	Orphan	36.34	325	6.18	65.72	64.22	-0.80
*BobHLH070*	*Bol029986*	IX	34.65	319	8.52	56.00	62.41	-0.62
*BobHLH071*	*Bol004925*	Orphan	56.20	504	5.44	50.17	77.08	-0.46
*BobHLH075*	*Bol011028*	Va	38.11	344	6.13	49.36	69.16	-0.73
*BobHLH093*	*Bol021577*	VII(a+b)	45.76	406	6.40	60.63	61.21	-0.78
*BobHLH094*	*Bol021588*	IX	32.94	302	6.67	57.82	53.97	-0.81
*BobHLH104*	*Bol020888*	XII	66.30	610	5.21	49.41	66.70	-0.53
*BobHLH105*	*Bol030777*	VIIIb	29.84	263	7.79	53.77	70.11	-0.75
*BobHLH109*	*Bol036767*	VII(a+b)	37.95	342	6.37	65.07	60.37	-0.72
*BobHLH111*	*Bol007664*	XII	50.41	457	6.14	57.04	64.46	-0.62
*BobHLH117*	*Bol023989*	VIIIc(1)	39.00	354	5.75	63.46	69.46	-0.57
*BobHLH124*	*Bol040072*	Ia	36.64	324	5.42	61.33	74.94	-0.75
*BobHLH126*	*Bol027107*	Ib(1)	25.08	219	9.27	54.41	70.68	-0.80
*BobHLH129*	*Bol041503*	XII	37.75	337	5.86	46.86	76.94	-0.55
*BobHLH147*	*Bol018679*	IVb	27.25	245	5.91	47.66	76.41	-0.69
*BobHLH148*	*Bol018659*	Ia	29.48	257	8.79	64.19	79.61	-0.64
*BobHLH149*	*Bol014189*	IVb	61.79	568	5.78	46.74	74.19	-0.49
*BobHLH152*	*Bol007436*	XI	39.69	349	5.50	66.58	73.50	-0.60
*BobHLH156*	*Bol037257*	II	36.43	328	4.93	55.42	58.57	-0.76
*BobHLH162*	*Bol044486*	III(a+c)	101.68	900	8.68	51.92	70.53	-0.56
*BobHLH172*	*Bol018435*	IIIb(1)	32.42	282	8.80	66.89	68.44	-0.69
*BobHLH173*	*Bol011442*	XII	34.63	310	6.15	73.35	77.71	-0.57
*BobHLH178*	*Bol032141*	III(d+e)	25.37	226	9.04	38.23	80.22	-0.67
*BobHLH202*	*Bol029707*	IVa	28.79	257	8.76	60.15	89.84	-0.30
*BobHLH207*	*Bol016340*	XI	34.26	298	6.22	66.88	78.56	-0.81
*BobHLH212*	*Bol009457*	VII(a+b)	27.15	246	5.19	60.28	63.46	-0.76
*BobHLH221*	*Bol004364*	IIIb(1)	40.51	359	5.08	63.80	74.40	-0.57

MW: Molecular weight, PL: Protein length, pI: Isoelectric point, GRAVY: Aliphatic index and grand average of hydropathicity, bHLH: Basic helix-loop-helix transcription factor.

## Supplementary Materials

The following are available online at https://www.mdpi.com/2073-4425/10/11/914/s1: Table S1. The genomic sequences of the 234 *BobHLH* genes; Table S2. The coding sequences of the 234 *BobHLH* genes; Table S3. The protein sequences of the 234 BobHLH proteins; Table S4. The characteristics of BobHLH family in *B. oleracea*; Table S5. Organ/tissue-expression FPKM values of *BobHLH* genes extracted from RNA-Seq data; Table S6. Chilling-expression FPKM values of *BobHLH* genes extracted from RNA-Seq data; Table S7. The characteristics of 234 BobHLH proteins in *B. oleracea*; Table S8. The details of *cis*-acting elements in the 2000 bp upstream of the *BobHLH* genes; Table S9. Subcellular localization prediction; Table S10. Primers of vector construction. Figure S1. The distribution of conserved motifs in each BobHLH protein; Figure S2. Sequence logos of BobHLH proteins domains; Figure S3. The distribution of exon-intron in *BobHLH* genes.
